# Fungal infection-related conditions and outcomes in severe COVID-19: a nationwide case-control study

**DOI:** 10.1186/s12879-024-10317-z

**Published:** 2024-12-18

**Authors:** Katsuya Maeshima, Ryo Yamamoto, Kazuki Matsumura, Daiki Kaito, Koichiro Homma, Kazuma Yamakawa, Takashi Tagami, Mineji Hayakawa, Takayuki Ogura, Atsushi Hirayama, Hideo Yasunaga, Junichi Sasaki, the J-RECOVER study group

**Affiliations:** 1 Department of Emergency and Critical Care Medicine, Keio University School of Medicine, 35 Shinanomachi, Shinjuku, Tokyo, 160-8582 Japan; 2 Department of Emergency and Critical Care Medicine, Osaka Medical and Pharmaceutical University, Osaka, Japan; 3 Department of Emergency and Critical Care Medicine, Nippon Medical School Musashikosugi Hospital, Kawasaki, Kanagawa Japan Japan; 4 Department of Emergency Medicine, Hokkaido University Hospital, Sapporo, Hokkaido Japan Japan; 5 Department of Emergency Medicine and Critical Care Medicine, Tochigi Prefectural Emergency and Critical Care Centre, Imperial Foundation Saiseikai Utsunomiya Hospital, Tochigi, Japan; 6 Public Health, Department of Social Medicine, Graduate School of Medicine, Osaka University, Osaka, Japan; 7 Department of Clinical Epidemiology and Health Economics, School of Public Health, The University of Tokyo, Tokyo, Japan

**Keywords:** COVID-19, Fungal infection, Steroid therapy, Mechanical ventilation, Risk factors

## Abstract

**Background:**

Fungal infections are significant complications of severe coronavirus disease 2019 (COVID-19). Although various risk factors for poor outcomes in patients with COVID-19 have been identified, clinical and treatment factors associated with fungal infections in patients with severe COVID-19 remain unclear. This study aimed to elucidate clinical factors associated with fungal infections during severe COVID-19 treatment.

**Methods:**

This was a post hoc analysis of the J-RECOVER study, a multicenter retrospective observational study involving patients with COVID-19 who required admission at 66 hospitals between January and September 2020. Inclusion criteria were ages ≥ 18 years, COVID-19 diagnosis with reverse-transcription polymerase chain reaction, and treatment with mechanical ventilation (MV). Patients who received antifungal drugs before MV were excluded. Potential predictors were identified through univariate analysis of patient and treatment characteristics between patients with- and those without fungal infection, which was defined as antifungal agent use for ≥ 5 days. To account for facility-specific data clustering, generalized estimating equations (GEE) were employed as adjusted analyses to calculate the relative risks of potentially associated factors. Two sensitivity analyses were performed with modified definitions for the two groups: patients who received antifungal drugs for ≤ 4 days were excluded, and fungal infection was re-defined as antifungal drug use for ≥ 14 days.

**Results:**

Among 4,915 patients in the J-RECOVER study, 559 adults with COVID-19 who required MV were included. Fungal infections occurred in 57 (10.2%) patients. Univariate analyses identified age, age ≥ 65 years, D-dimer level, remdesivir use, steroid use, and duration of steroid therapy as potential predictors of fungal infections. Multivariate analysis using GEE on these six factors revealed that only the duration of steroid use was significantly associated with an increased risk of fungal infection (odds ratio [OR] for a day increase: 1.01; 95% confidence interval [CI]: 1.00–1.01; *p* < 0.001). The two sensitivity analyses similarly showed that the duration of steroid use was associated with fungal infection (odds ratio for a day increase: 1.01; 95% CI: 1.00–1.01; *p* < 0.001 for both).

**Conclusions:**

In patients with severe COVID-19 requiring MV, each additional day of steroid use was associated with prolonged use of antifungal medications for ≥ 5 days.

**Supplementary Information:**

The online version contains supplementary material available at 10.1186/s12879-024-10317-z.

## Background

The clinical outcomes of coronavirus disease 2019 (COVID-19) have improved with the development of pharmacological therapy and critical care management. Severe acute respiratory syndrome coronavirus 2 has also changed in pathogenicity over time, resulting in less severe clinical outcomes, such as a shorter length of hospital stay and reduced mortality [1, 2]. A recent study reported that the mortality rate per 1,000 patients decreased from 4.48 in the first wave to 0.67 in the fifth wave [3].

However, some populations still suffer from severe pulmonary and organ dysfunction due to COVID-19. Some observational studies have suggested the risks of exacerbation, including hematologic malignancies, diabetes mellitus, chronic kidney disease, and co-infection with bacterial and/or fungal pathogens [4–8]. Particularly, patients with fungal infections during treatment for COVID-19 have been reported to more frequently require intensive care and suffer from mortality. Thus, minimizing such additional risks is another target for the management of COVID-19 [9].

While some underlying conditions that predispose patients to fungal infections are similar to those associated with unfavorable outcomes after COVID-19, modifiable risk factors for fungal infections also exist. A multicenter cohort study on patients with COVID-19 managed with mechanical ventilation (MV) found that the concomitant use of anti-interleukin (IL)−6 and steroids increased the incidence of fungal infections [10]. Additionally, another cohort study involving patients with COVID-19 in an intensive care unit (ICU) suggested that long-term and/or high-dose steroids could be risk factors for fungal coinfection [11]. Accordingly, this study aimed to elucidate the clinical factors associated with the incidence of fungal infection during COVID-19 treatment, focusing on potentially avoidable treatments.

## Methods

### Study design and setting

This was a secondary analysis of the J-RECOVER study [12]. The J-RECOVER study was conducted to investigate the clinical characteristics of COVID-19 and included patients diagnosed with moderate-to-severe COVID-19 (requiring oxygen and/or ICU admission) at 66 institutions in Japan between January and September 2020, during which two waves of increased number of patients with COVID-19 occurred. The J-RECOVER study included patients diagnosed with COVID-19 using a positive reverse transcription polymerase chain reaction (RT-PCR). The J-RECOVER study was conducted after obtaining approval from the institutional review board (IRB) to conduct research on human participants at participating institutions. The IRB of the Keio University School of Medicine approved this study on human participants (application number: 20200317). As the data in the study were anonymous, the requirement for informed consent was waived.

### Study population

The inclusion criteria for this study were ages ≥ 18 years, COVID-19 diagnosis with RT-PCR, and MV usage. Patients who met the criteria were included in the study. Patients who received antifungal drugs before the initiation of MV were excluded.

### Data collection and definitions

In the J-RECOVER study, data were collected from medical records and diagnosis procedure combinations at each participating facility, which is a method of calculating medical costs based on the diseases and details of medical treatment as defined by the Japanese Ministry of Health, Labour and Welfare; the format is standardized in Japan and recorded by physicians [13]. These data included the diagnosis at admission, comorbidities, Hugh-Jones classification [14], New York Heart Association (NYHA) classification documented at admission [15], dates and amounts of drugs, and discharge summaries. In this study, we investigated the Charlson comorbidity index [16], related comorbidities, smoking, Hugh-Jones classification, NYHA classification, consciousness, respiratory status, and blood test results as the background of the patient’s condition.

Data on antifungal drugs, steroids, remdesivir, and tocilizumab were also retrieved. However, the types of antifungal drugs were not available in the database. The list of administered steroids, except for topical drugs, was presented in the supplementary Table 1, which included oral and intravenous medications. The duration of steroid use was also assessed. Moreover, the number of days from hospital arrival to MV and from the initiation of MV to the administration of antifungal drugs were calculated. Data on in-hospital mortality, length of hospital stay, and ventilator- and ICU-free days up to 28 days after admission were also obtained.

### Outcome measures

The primary outcome was the fungal infection, which was defined as the administration of antifungal drugs for ≥ 5 consecutive days: antifungal drugs should have been discontinued within 5 days if culture results returned negative for fungus, which generally takes less than 5 days. Culture results for fungal infections were not available in the database.

### Data preparation and sample size estimation

The sample size for implementing multivariate logistic regression analysis with five possible risk factors was estimated to be at least 50 patients with fungal infections. Given that a quarter of the patients with COVID-19 who were treated with MV would have fungal infections based on previous studies, at least 200 patients with COVID-19 needed to be included in this study.

### Statistical analysis

Patient characteristics are presented as number (%) or median (interquartile range) and were compared between patients with- and those without fungal infection. Univariate analysis was performed for each factor of patient characteristics and clinical consequences using Chi-squared tests or Mann–Whitney U tests, as appropriate, and the standardized mean difference was calculated to show substantial differences in each variable. Then, clinical factors with a substantial difference (standardized mean difference ≥ 0.3) were considered potentially relevant factors. Generalized estimating equation (GEE) models were used to investigate the factors associated with fungal infection, in which the potentially relevant factors obtained in the univariable analyses were entered.

We performed two sensitivity analyses. First, patients with ≤ 4 days of antifungal drug use, who were originally included as those without fungal infection, were excluded, and GEE analyses were repeated with the same variables as the original model. Second, fungal infection was re-defined as administration of antifungal drugs for ≥ 14 consecutive days, and GEE analyses were performed.

Statistical analyses were performed using the Statistical Package for the Social Sciences software (version 29.0; IBM Corp., Armonk, NY, USA) and Microsoft Excel (Microsoft, Redmond, WA, USA).

## Results

### Characteristics of patients

**Fig. 1:**
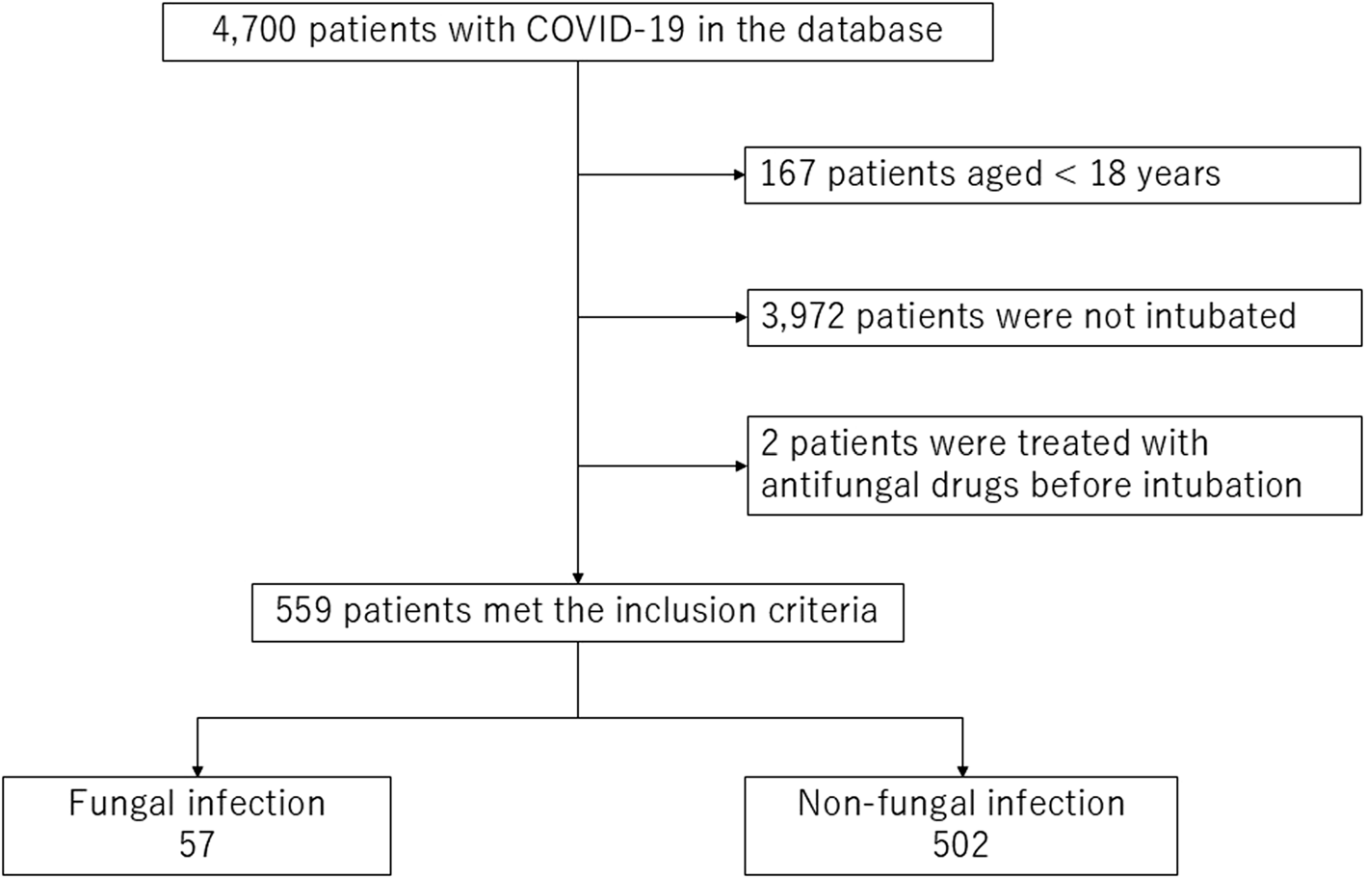
Patient flow diagram. A total of 4,915 patients with coronavirus disease 2019 (COVID-19) were included in the J-RECOVER database. A total of 559 patients met the inclusion criteria. Of these patients, 57 fulfilled the definition of fungal infection and 502 were classified as having non-fungal infections

Among the 4,700 patients in the J-RECOVER study, 559 adult patients diagnosed with COVID-19 were treated with MV and met all inclusion criteria. The patients included in the analysis were categorized into 57 and 502 patients with- and without fungal infections, respectively (Fig. 1).

 Patient characteristics are shown in Table 1. The median age was higher in patients with fungal infection compared to those without (72.0 [63.5–77.5] vs. 66.0 [55.0–75.0]). The frequency of steroid use was higher in patients with fungal infections (48 [84.2%] vs. 281 [56.0%]), whereas dexamethasone was administered similarly to patients with- and those without fungal infections. The duration of steroid therapy was longer in patients with fungal infection (16.0 [4.5–32.0] vs. 2.0 [0.0–10.0] days).

**Table 1:** Characteristics of patients with and without fungal infection

	With fungal infection		Without fungal infection		Standardzed Difference	*p* value
Case	57		502			
Age, years, median (IQR)	72.0	(63.5-77.5)	66	(55.0-75.0)	0.374	0.010
Age, ≥65, n(%)	41	(71.9%)	270	(53.8%)	0.382	0.011
Sex,male, n (%)	43	(75.4%)	398	(79.3%)	0.092	0.496
Charlson index, median (IQR)	0	(0-1)	0	(0-1)	0.040	0.689
Comorbidity, n (%)						
Chronic lung disease	0	(0.0%)	1	(0.2%)	0.063	1.000
Diabetes	14	(24.6%)	139	(27.7%)	0.071	0.754
HIV/AIDS	0	(0.0%)	1	(0.2%)	0.063	1.000
Malignancy	0	(0.0%)	12	(2.3%)	0.221	0.622
Chronic kidney disease	5	(8.8%)	15	(3.0%)	0.248	0.043
Social history						
Smoking, n (%)	11	(33.3%)	119	(41.0%)	0.160	0.457
Chronic cardiopulmonary status						
Hugh-Jones classification, > III, n (%)	9	(15.8%)	79	(15.7%)	0.001	1.000
NYHA functional classification, > II, n (%)	1	(1.8%)	5	(1.0%)	0.065	0.477
Status on hospital arrival						
GCS, median (IQR)	14.5	(5.5-15.0)	15	(8.0-15.0)	0.163	0.094
Respiratory rate, /min, median (IQR)	21	(15.0-26.0)	22	(18.0-26.0)	0.149	0.202
Oxygen requirement, ≥ 4 L/ min, n (%)	23	(40.4%)	206	(41.0%)	0.014	1.000
SOFA, hemodynamic score, median (IQR)	0	(0-0)	0	(0-0)	0.144	0.338
Blood test at time of admission, median (IQR)						
WBC, 10^3^ /μL	7.2	(5.1-9.6)	7	(5.4-10.4)	0.009	0.848
CRP, mg/dL	13.3	(6.1-25.8)	10.8	(5.7-16.7)	0.186	0.041
D-dimer, μg/dL	2.6	(1.3-9.3)	1.7	(0.9-4.3)	0.314	0.036
Lactate, ≥ 2 mmol/L, n (%)	7	(12.3%)	55	(15.2%)	0.041	0.823
Status at the time of intubation						
PF ratio, mmHg, median (IQR)	142	(106.0-198.9)	162	(121.3-224.5)	0.239	0.087
SOFA, hemodynamic score, median (IQR)	0	(0-0)	0	(0-0)	0.044	0.598
Lactate, ≥ 2 mmol/L, n (%)	6	(12.5%)	46	(10.3%)	0.046	0.620
Medications, n (%)						
Remdesivir, n (%)	7	(12.3%)	130	(25.9%)	0.352	0.023
Tocilizumab, n (%)	10	(17.5%)	45	(9.0%)	0.255	0.056
Dexamethasone, n (%)	9	(15.8%)	107	(21.3%)	0.143	0.391
Steroid, n (%)	48	(84.2%)	281	(56.0%)	0.648	<0.001
Days during any steroid therapy, median (IQR)	16.0	(4.5-32.0)	2.0	(0-10.0)	0.805	<0.001
Days from arrival to intubation, median (IQR)	0	(0-2)	0	(0-1)	0.024	0.896
Days from symptom onset to intubation, median (IQR)	8	(5-10)	8	(6-11)	0.208	0.087
Days from mechanical ventilation to antifungal drugs, median (IQR)	15.0	(8.0-21.5)	15	(8.0-19.5)	0.069	0.804

*COVID-19* Coronavirus disease 2019, *IQR *Interquartile range, *HIV *Human immunodeficiency virus, *AIDS *Aquired immune deficiency syndrome, *NYHA *New York Heart Association, *GCS *Glasgow Coma Scale, *SOFA *Sequential Organ Failure Assessment, *WBC* White blood cell count, *CRP *C-reactive protein, *PF ratio *ratio of partial pressure of oxygen and fraction of inspired oxygen

**Fig. 2:**
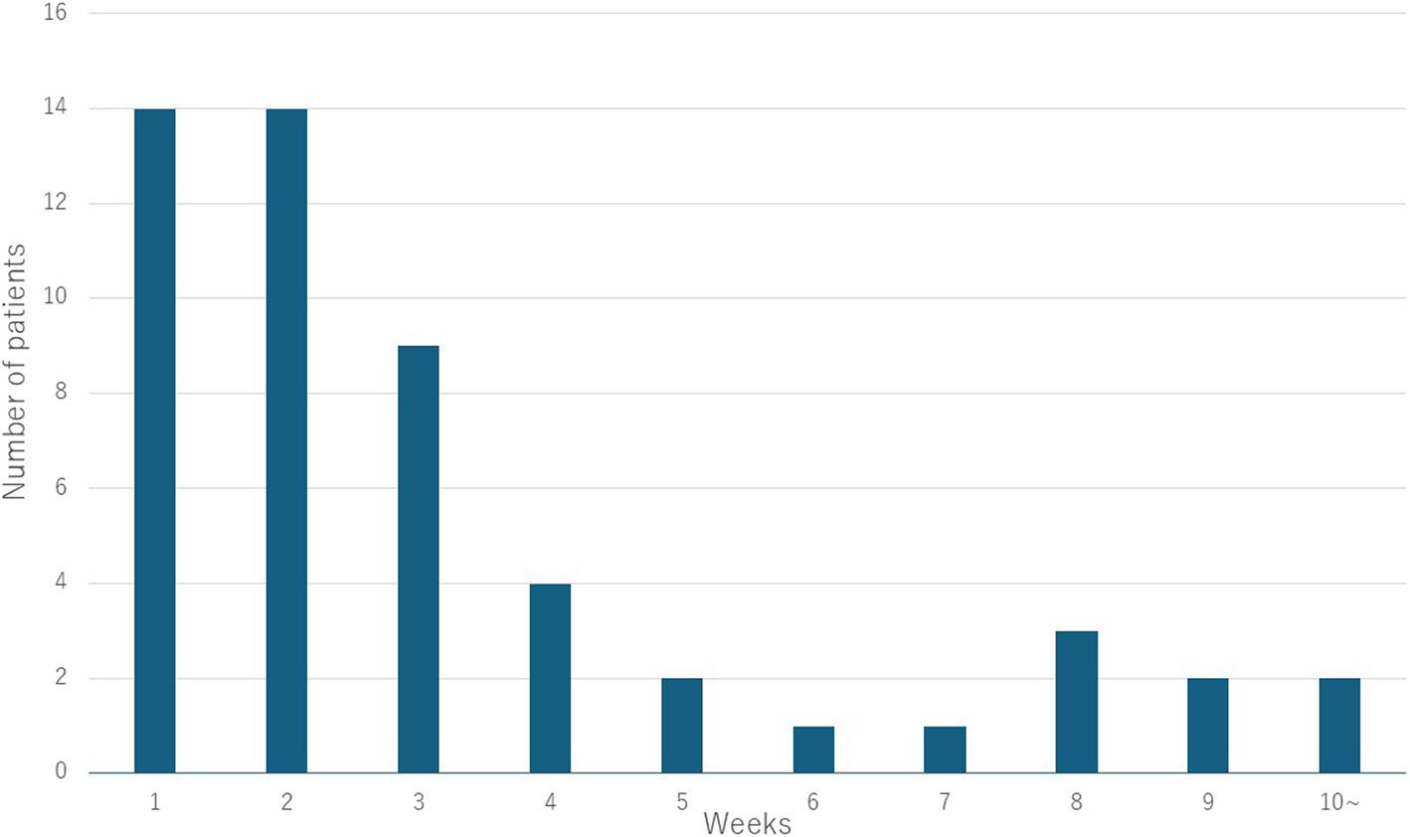
Duration of antifungal drug administration in patients with fungal infection. Histograms show the number of cases that met the definition of fungal infection for each period of antifungal drug administration. Most treatments were administered for 1–3 weeks, with 2 weeks being the most common

Regarding the duration of antifungal treatment, most patients were treated for 1–3 weeks, with a 2-week administration being the most frequent (Fig. 2).

 Higher in-hospital mortality, longer hospital stays, and shorter ventilator- and ICU-free days up to day 28 were observed in patients with fungal infections than in those without fungal infections (Table 2).

**Table 2:** Outcomes of patients with and without fungal infection

	With fungal infection		Without fungal infection		*p* value
Mortality, n(%)	32	(56.1%)	112	(22.3%)	<0.001
Length of hospital stay, median(IQR)	36	(26.5-72.5)	20	(11.0-31.0)	<0.001
Ventilator-free days to day 28 after intubation, median(IQR)	11.5	(0-20.3)	25	(18.0-27.0)	<0.001
ICU-free days to day 28 after admission, median (IQR)	0	(0-0)	12	(0-19.0)	<0.001

*IQR I*nterquartile range, *ICU *Intensive care unit

### Predictive factors for fungal infection

 Age, Age ≥ 65 years old, D-dimer, remdesivir, steroid, and days of steroid therapy were significantly associated with prolonged use of antifungal drug for ≥ 5 days based on univariate analyses (Table 1); therefore, they were entered into the GEE model. The GEE model showed the duration of steroid treatments as the only predictor for fungal infection in patients with severe COVID-19 (odds ratio [OR] in 1-day increase of steroid use, 1.01; 95% confidence interval [CI], 1.004–1.01; *p* < 0.001; Table 3).

**Table 3:** Odds ratio of potential predictors for fungal infection

Variable	Odds ratio	95% confidence interval	*p* value
Age	1.00	1.00-1.00	0.975
Age, ≥65	1.05	0.96-1.14	0.275
D-dimer	1.00	1.00-1.00	0.079
Remdesivir	0.95	0.89-1.02	0.129
Steroid	0.98	0.92-1.05	0.575
Days during any steroid therapy	1.01	1.00-1.01^a^	<0.001

^a^Actual interval was 1.004 to 1.01

 Sensitivity analyses after excluding patients with 1–4 days of antifungal medication use and redefining fungal infection as antifungal treatment ≥ 14 days also revealed that the days of steroid use was a predictor of fungal infection (OR in a 1-day increase of steroid use, 1.01; 95% CI, 1.004–1.01; *p* < 0.001 in both sensitivity analyses; Tables 4 and 5). Moreover, another sensitivity analysis incorporating broad-spectrum antibiotic use as an additional variable showed similar results (OR in a 1-day increase of steroid use, 1.05; 95%CI, 1.02–1.09).

**Table 4:** Sensitivity analysis in patients without less than five days of antifungal medication

Variable	Odds ratio	95% confidence interval	*p* value
Age	1.00	1.00-1.00	0.911
Age, ≥65	1.05	0.96-1.14	0.300
D-dimer	1.00	1.00-1.00	0.084
Remdesivir	0.95	0.89-1.02	0.138
Steroid	0.99	0.92-1.05	0.691
Days during any steroid therapy	1.01	1.00-1.01^a^	<0.001

^a^Actual interval was 1.004 to 1.01

**Table 5:** Sensitivity analysis with re-defining fungal infection

Variable	Odds ratio	95% confidence interval	*p* value
Age	1.00	1.00-1.00	0.938
Age, ≥65	1.03	0.96-1.11	0.407
D-dimer	1.00^a^	1.00-1.00	0.042
Remdesivir	0.99	0.94-1.03	0.560
Steroid	0.98	0.93-1.03	0.413
Days during any steroid therapy	1.01	1.00-1.01^b^	<0.001

Fungal infection was re-defined as antifungal treatment ≥14 days

^a^Actual odds ratio was 1.0020 (95% Confidence Interval was 1.0001 to 1.0040)

^b^Actual interval was 1.004 to 1.01

## Discussion

This study revealed an association between steroid use duration and fungal infections in patients with severe COVID-19 requiring MV. A day-long increase in steroid use showed a modest association with prolonged use of antifungal drug for ≥ 5 days, defined as fungal infection in this study, and this association remained consistent in two sensitivity analyses that adjusted for the definitions of fungal infection.

The pathophysiological reasons for these results can be explained with basic studies. Prolonged steroid use impairs the migration of macrophages and neutrophils to the infection site by decreasing or inhibiting the release of chemotactic factors. As the fact that the immune system against fungi includes phagocytosis by macrophages and cytotoxicity by polymorphonuclear leukocytes formed by chemotactic neutrophils is well known [17], steroids use might have contributed to prolonged use of antifungal drugs for clinically suspected invasive fungal infection. Moreover, dose- and duration-dependent immunosuppressive effects of steroids have been revealed in various studies [18–20], which could be shown as a risk increment by a 1-day increase in steroid use.

The observed odds ratio of 1.01 for each additional day of steroid use indicates a modest incremental risk (e.g., an OR of 1.1 for a 10-day increase). While the clinical implications should be carefully interpreted, even a small risk increment might still be clinically relevant because steroids are used more than often in severe COVID-19. While a 10-day use of dexamethasone for moderate-to-severe COVID-19 has been shown to decrease mortality [21], a prolonged regimen of steroids is still used worldwide [22, 23]. As increased susceptibility to fungal infections is associated with daily steroid use, only a validated regimen should be used. Furthermore, trial use of steroids for a few days should be avoided. In the current study, 327 of the 559 included patients (58%) and 16 of the 57 patients with fungal infection (28%) received steroids only for 5 days or less. As trial use of short-duration steroid regimens has now shown clinical benefits [24], physicians should be aware that fungal infections could occur even without long-term treatment.

It should be emphasized that microbiological proof of fungal infection with a positive culture of fungus was not available in this study and therefore the diagnosis unmet the European Organization for Research and Treatment of Cancer and the Mycoses Study Group Education and Research Consortium consensus definitions of invasive fungal diseases. However, the fungal infection was defined as equal to or longer than 5 days of antifungal drug use, considering that antifungal drugs would highly likely be discontinued within 5 days when the fungal culture was negative [25, 26]. Therefore, the current study at least indicated that an increase in duration of steroid use was associated with prolonged antifungal drug administration with clinically suspected invasive fungal infection.

This study had some limitations. First, its retrospective design might have introduced biases due to unmeasured confounding factors, such as liver disease, post-transplant status, solid and/or hematological malignances, and several biomarkers including IL-6 and tumor necrosis factor α. Second, as microbiological, serological, and radiological data and autopsy findings for fungal infections are lacking, the association between longer duration of steroid use and higher risks for invasive fungal infection was not confirmed in the current study and should be validated with pathological data in another study. Third, the generalizability of our findings to recent COVID-19 subtypes might be limited, given the evolution of viral pathogenicity and the widespread implementation of vaccination programs. Future prospective studies incorporating cultural results should be conducted to obtain such information. Third, this study was conducted in 2020, during the early phases of the COVID-19 pandemic. Treatment protocols have evolved, potentially limiting the generalizability of our findings to the current practice. Replication with recent cohorts would help validate our results in the context of the current COVID-19 management strategies.

## Conclusions

This study revealed an association between steroid use duration and prolonged antifungal drug uses for clinically suspected invasive fungal infections in patients with severe COVID-19 requiring MV. A daily increase in steroid use was associated with prolonged use of antifungal medications for ≥ 5 days.

## Supplementary Information

Supplementary Material 1.

## Data Availability

The data used in this study were obtained from the J-RECOVER study group. There were certain restrictions on access to data that were permitted for use in this study. These data are not publicly available but may be accessed upon reasonable request and with a license from the J-RECOVER study group.

## Abbreviations

CI: Confidence Interval
COVID-19: Coronavirus disease 2019
GEE: Generalized estimating equations
ICU: Intensive care unit
IL: Interleukin
IRB: Institutional Review Board
J-RECOVER: Japanese multicenter research of COVID-19 by assembling real-world data
MV: Mechanical ventilation
NYHA: New York Heart Association
OR: Odd ratio
RT-PCR: Reverse transcription polymerase chain reaction

## References

[1] Iuliano AD, Brunkard JM, Boehmer TK, Peterson E, Adjei S, Binder AM, et al. Trends in Disease Severity and Health Care utilization during the early omicron variant period compared with previous SARS-CoV-2 high transmission periods – United States, December 2020-January 2022. MMWR Morb Mortal Wkly Rep. 2022;71:146–52. 35085225 10.15585/mmwr.mm7104e4 PMC9351529

[2] Shi M, Fiori K, Kim RS, Gao Q, Umanski G, Thomas I, et al. Social needs assessment and linkage to community health workers in a large urban hospital system. J Prim Care Community Health. 2023;14: 21501319231166918. 37083206 10.1177/21501319231166918 PMC10126704

[3] Nab L, Parker EPK, Andrews CD, Hulme WJ, Fisher L, Morley J, et al. Changes in COVID-19-related mortality across key demographic and clinical subgroups in England from 2020 to 2022: a retrospective cohort study using the OpenSAFELY platform. Lancet Public Health. 2023;8:e364-77. 10.1016/S2468-2667(23)00079-8. 37120260 10.1016/S2468-2667(23)00079-8 PMC10139026

[4] Arcani R, Colle J, Cauchois R, Koubi M, Jarrot PA, Jean R, et al. Clinical characteristics and outcomes of patients with haematologic malignancies and COVID-19 suggest that prolonged SARS-CoV-2 carriage is an important issue. Ann Hematol. 2021;100:2799–803. 10.1007/s00277-021-04656-z. 34518918 10.1007/s00277-021-04656-z PMC8437431

[5] Guo W, Li M, Dong Y, Zhou H, Zhang Z, Tian C, et al. Diabetes is a risk factor for the progression and prognosis of COVID-19. Diabetes Metab Res Rev. 2020;36: e3319. 10.1002/dmrr.3319. 32233013 10.1002/dmrr.3319 PMC7228407

[6] D’Marco L, Puchades MJ, Romero-Parra M, Gimenez-Civera E, Soler MJ, Ortiz A, et al. Coronavirus disease 2019 in chronic kidney disease. Clin Kidney J. 2020;13:297–306. 10.1093/ckj/sfaa104. 32699615 10.1093/ckj/sfaa104 PMC7367105

[7] Adalbert JR, Varshney K, Tobin R, Pajaro R. Clinical outcomes in patients co-infected with COVID-19 and Staphylococcus aureus: a scoping review. BMC Infect Dis. 2021;21:985. 10.1186/s12879-021-06616-4. 34548027 10.1186/s12879-021-06616-4 PMC8453255

[8] Rovina N, Koukaki E, Romanou V, Ampelioti S, Loverdos K, Chantziara V, et al. Fungal infections in critically ill COVID-19 patients: inevitabile malum. J Clin Med. 2022;11: 2017. 10.3390/jcm11072017. 35407625 10.3390/jcm11072017 PMC8999371

[9] Alshrefy AJ, Alwohaibi RN, Alhazzaa SA, Almaimoni RA, AlMusailet LI, AlQahtani SY, et al. Incidence of bacterial and fungal secondary infections in COVID-19 patients admitted to the ICU. Int J Gen Med. 2022;15:7475–85. 10.2147/IJGM.S382687. 36187162 10.2147/IJGM.S382687 PMC9518678

[10] Gangneux JP, Dannaoui E, Fekkar A, Luyt CE, Botterel F, De Prost N, et al. Fungal infections in mechanically ventilated patients with COVID-19 during the first wave: the French multicentre MYCOVID study. Lancet Respir Med. 2022;10:180–90. 34843666 10.1016/S2213-2600(21)00442-2 PMC8626095

[11] Negm EM, Mohamed MS, Rabie RA, Fouad WS, Beniamen A, Mosallem A, et al. Fungal infection profile in critically ill COVID-19 patients: a prospective study at a large teaching hospital in a middle-income country. BMC Infect Dis. 2023;23:246. 10.1186/s12879-023-08226-8. 37072718 10.1186/s12879-023-08226-8 PMC10111294

[12] Tagami T, Yamakawa K, Endo A, Hayakawa M, Ogura T, Hirayama A, et al. Japanese multicenter research of COVID-19 by assembling real-world data: a study protocol. Ann Clin Epidemiol. 2022;4:92–100. 10.37737/ace.22012. 38504944 10.37737/ace.22012 PMC10760490

[13] Hayashida K, Murakami G, Matsuda S, Fushimi K. History and profile of diagnosis Procedure Combination (DPC): development of a real data collection system for acute inpatient care in Japan. J Epidemiol. 2021;31:1–11. 10.2188/jea.JE20200288. 33012777 10.2188/jea.JE20200288 PMC7738645

[14] Hugh-Jones P, Lambert AV. A simple standard exercise test and its use for measuring exertion dyspnoea. Br Med J. 1952;1:65–71. 10.1136/bmj.1.4749.65. 14896031 10.1136/bmj.1.4749.65 PMC2022477

[15] Bennett JA, Riegel B, Bittner V, Nichols J. Validity and reliability of the NYHA classes for measuring research outcomes in patients with cardiac disease. Heart Lung. 2002;31:262–70. 10.1067/mhl.2002.124554. 12122390 10.1067/mhl.2002.124554

[16] Charlson ME, Pompei P, Ales KL, MacKenzie CR. A new method of classifying prognostic comorbidity in longitudinal studies: development and validation. J Chronic Dis. 1987;40:373–83. 10.1016/0021-9681(87)90171-8. 3558716 10.1016/0021-9681(87)90171-8

[17] Latgé JP. Aspergillus fumigatus and aspergillosis. Clin Microbiol Rev. 1999;12:310–50. 10.1128/CMR.12.2.310. 10194462 10.1128/cmr.12.2.310 PMC88920

[18] Widdifield J, Bernatsky S, Paterson JM, Gunraj N, Thorne JC, Pope J, et al. Serious infections in a population-based cohort of 86,039 seniors with rheumatoid arthritis. Arthritis Care Res. 2013;65:353–61. 10.1002/acr.21812. 10.1002/acr.21812 22833532

[19] Marr KA, Carter RA, Boeckh M, Martin P, Corey L. Invasive aspergillosis in allogeneic stem cell transplant recipients: changes in epidemiology and risk factors. Blood. 2002;100:4358–66. 10.1182/blood-2002-05-1496. 12393425 10.1182/blood-2002-05-1496

[20] Lewis RE, Kontoyiannis DP. Invasive aspergillosis in glucocorticoid-treated patients. Med Mycol. 2009. 10.1080/13693780802227159. 47;Suppl 1, Issue Supplement_1:S271–81. 18654923 10.1080/13693780802227159

[21] RECOVERY Collaborative Group, Horby P, Lim WS, Emberson JR, Mafham M, Bell JL, et al. Dexamethasone in hospitalized patients with Covid-19. N Engl J Med. 2021;384:693–704. 10.1056/NEJMoa2021436. 32678530 10.1056/NEJMoa2021436 PMC7383595

[22] Coutinho AE, Chapman KE. The anti-inflammatory and immunosuppressive effects of glucocorticoids, recent developments and mechanistic insights. Mol Cell Endocrinol. 2011;335:2–13. 10.1016/j.mce.2010.04.005. 20398732 10.1016/j.mce.2010.04.005 PMC3047790

[23] Villar J, Confalonieri M, Pastores SM, Meduri GU. Rationale for prolonged corticosteroid treatment in the acute respiratory distress syndrome caused by coronavirus disease 2019. Crit Care Explor. 2020;2: e0111. 10.1097/CCE.0000000000000111. 32426753 10.1097/CCE.0000000000000111 PMC7188431

[24] Li Y, Li J, Ke J, Jiao N, Zhu L, Shen L, et al. Adverse outcomes associated with corticosteroid use in critical COVID-19: a retrospective multicenter cohort study. Front Med (Lausanne). 2021;8: 604263. 10.3389/fmed.2021.604263. 33634148 10.3389/fmed.2021.604263 PMC7900536

[25] Reisner BS, Woods GL. Times to detection of bacteria and yeasts in Bactec 9240 blood culture bottles. J Clin Microbiol. 1999;37:2024–6. 10.1128/JCM.37.6.2024-2026.1999. 10325369 10.1128/jcm.37.6.2024-2026.1999 PMC85018

[26] Ziegler R, Johnscher I, Martus P, Lenhardt D, Just HM. Controlled clinical laboratory comparison of two supplemented aerobic and anaerobic media used in automated blood culture systems to detect bloodstream infections. J Clin Microbiol. 1998;36(3):657–61. 10.1128/JCM.36.3.657-661.1998. 9508291 10.1128/jcm.36.3.657-661.1998 PMC104604

